# Continuous approximation and GIS-enhanced design optimization of feeder bus networks along rail corridors

**DOI:** 10.1371/journal.pone.0318616

**Published:** 2025-05-23

**Authors:** Jiahe Liu, Rui Song

**Affiliations:** 1 Key Laboratory of Transport Industry of Big Data Application Technologies for Comprehensive Transport, Beijing Jiaotong University, Beijing, China; 2 Beijing Traffic Management Bureau, Beijing, China; Istanbul Technical University: Istanbul Teknik Universitesi, TÜRKIYE

## Abstract

As urbanization accelerates, urban public transportation systems are tasked with meeting the growing demand for travel. According to statistics, the regional rail transit network currently handles 60% of the passenger volume for trips originating from around the stations, and this is projected to increase by over 20% within the next decade. However, the current feeder bus services suffer from insufficient coverage, leading to inconvenience for passengers and impacting the overall efficiency of the transportation system. To enhance the public transit service coverage along a rail transit corridor, this study designs feeder bus services to connect the rail stations. A theoretical trunk-feeder transit design problem is firstly solved to furnish the initial feeder bus line designs. The objective is to minimize total generalized system cost with respect to the density of feeder bus routes and their service frequencies. The idealized design of the optimal bus route density function is then discretized into specific locations and fine-tuned with Geographic Information System (GIS) tool. Considering local conditions like road network and demographic information, a four-step adjustment strategy is proposed to furnish the implementation-ready design of feeder bus services. Numerical results show the final design renders at least 3.5% saving in total system cost. This study illustrates how the state-of-the-art theories of transit design can be applied into practice and highlights the connection between theoretical studies and practical application.

## 1 Introduction

In urban public transit systems, light rail lines always operate as main arteries, and their service areas can be expanded through feeder bus services, which is the core focus of this study. A light rail line currently under construction in a foreign country is a main line that spans a 26.3-kilometer corridor between regions A and B. This line is expected to transport 69,000 passenger trips per day (according to the local transit administration’s 2022 data) [1]. However, the existing 12 bus routes operating in and around the corridor are not optimally designed to support the anticipated passenger volume, indicating a need for an enhanced feeder service network.

The primary objective of this research is to address this need by designing an efficient feeder bus service that minimizes total system costs while maximizing coverage and accessibility along the rail transit corridor. By employing a theoretical trunk-feeder transit design model and integrating it with Geographic Information System (GIS) tools, we aim to develop a practical and implementable feeder bus service design. This design will consider the density of feeder bus routes and their service frequencies, ultimately leading to a proposed network that is expected to reduce overall system costs by at least 3.5%.

The significance of this study lies in its approach to bridging the gap between theoretical transit design models and practical application, offering a scalable solution that can be adapted to various urban environments. The expected outcome is an optimized feeder bus network that not only meets the growing demand along the light rail corridor but also serves as a model for future transit system expansions and improvements. The literature on the feeder-bus system design problem can be divided to two categories according to the modelling methodologies: discrete optimization models and continuous approximation models. Table 1 summarizes the characteristics of representative discrete and continuous models. The discrete models, with discrete inputs of demand and decision variables, can be well built to reflect realistic conditions, e.g., network structure and geographic features.For instance, Kuah and Perl (1987, 1989)[2,3] proposed a representative discrete model to design a set of feeder-bus routes and determine the frequency on each route. The demand is assumed to be concentrated at given nodes (rail stations and bus stops) and the objective is to minimize total operator and user costs. Similar modeling framework is adopted by many following researchers (Deng et al. 2013, 2020[4,5]; Kuan et al. 2006[6]; Mohaymany and Gholami 2010[7]; Shrivastava and O’Mahony 2006, 2007[8,9]). To account for broader demand distribution in accordance with traffic analysis zones (TAZ), Chien and Yang (2000)[10], Xiong et al. (2013)[11] modeled zone-based feeder-bus system design in a grid network without the need of pre-specifying candidate stops/lines. As opposed to previous specific demand pattern (i.e., many-to-one distribution) for commuting patrons, Szeto and Wu (2011)[12], Charisis et al. (2018)[13] considered more general many-to-many demand pattern in their models, of which the optimization is extended to a multi-objective problem. To further account for the response of demand to network design, Szeto and Jiang (2014)[14] proposed a bi-level model with the upper-level problem determining feeder bus system design and the lower-level problem conducting transit assignment.

**Table 1:** Representative studies on feeder bus design problem.

Literature	Objective function	Route design parameter	Input demand	Network structure
Discrete models				
Kuah and Perl (1989) [3]	Minimize total cost, including operator and user costs	0-1 dummy variables to determine stops on each route	Hourly demand per stop (Many-to-one)	Topological network
Kuan et al. (2006) [6]		An integer string with each substring being a sequence of stops in a route	Hourly demand per stop (Many-to-one)	
Chien and Yang (2000) [10]		Route location represented by link and node incident matrices	Demand density by zone (Many-to-one)	Irregular grid street network
Szeto and Wu (2011) [12]	Minimize the weighted sum of the number of transfers and total passengers’ travel time	0-1 dummy variables to determine stops on each route	Demand matrix by node (Many-to-many)	Real network
Szeto and Jiang (2014) [14]	Minimize the sum of transfer passengers; minimize the sum of the total in-vehicle travel and stop times and total waiting time	0-1 dummy variables to determine stops on each route	Demand matrix by node (Many-to-many)	
Continuum Approximation models				
Hurdle (1973) [27]	Minimize total cost, including operator and user costs	Density of feeder lines	Demand density function (Many-to-one)	Parallel bus routes
Wirasinghe (1980) [28]		Feeder-bus routes density, inter-station spacing of rail line	Demand density function (Many-to-one)	
Kuah and Perl (1988) [29]		Bus-route spacing, bus-stop spacing	Demand density function (Many-to-one)	
Chien and Schonfeld (1998) [30]		Rail line length, rail station spacing, bus stop spacing, bus route spacing	Demand density by zone (Many-to-many)	
Fan and Mei (2017) [31]		Trunk station density, feeder bus line density, stop density	Boarding and alighting density functions along the trunk line (Many-to-many)	
Sivakumaran et al. (2014) [32]		Bi-direction trunk line spacings, trunk station spacing, vehicle headway	Uniformly distributed demand	Parallel bus routes within a grid trunk system
Hugo Badia, Erik Jenelius (2021) [34]		line spacing, stop spacing, headway, and other geometrical	Demand density by zone (Many-to-many)	Parallel bus routes
Li Xin,Dai Zhang (2022) [35]		Headway, Line Spacing, Stop Spacing	Demand density by zone (Many-to-many)	Parallel bus routes
Jingyuan Qiao (2023) [36]		Bus line spacing, station spacing, and headway of bimodal transit systems.	Demand density by zone (Many-to-many)	Parallel bus routes

The discrete models were often constructed as mixed integer non-linear programs, which are NP-hard and mainly rely on (meta-) heuristic algorithms. For instance, a feeder route generation algorithm was developed by Baaj and Mahmassani (1995)[15] using node selection and insertion strategies. Based on this, Shrivastav and Dhingra (2001)[16] further added constraints on restricting too short routes near railway station. Tabu search strategy was employed by Martins and Pato (1998)[17]. Among these heuristics, genetic algorithm appeared to be the most popular one in the literature (Deng et al. 2013, 2020 [4,5]; Kuan et al. 2006 [6]; Shrivastava and O’Mahony 2006, 2007 [8,9]; Xiong et al. 2013 [11]; Szeto and Wu 2011 [12]; Charisis et al. 2018 [13]).However, these algorithms cannot guarantee the solution quality for large-scale network design problem, which requires a huge number of variables and input parameters. The solution finding efficiency also deteriorates fast with the problem scale, which does not allow in-depth parametric analysis. Thus, no generic relationships can be unveiled between the optimal solutions and input parameters. In a nutshell, the discrete models are time consuming and data hungry, on the one hand. On the other hand, the outputs of these models are oftentimes limited to particular cases/scenarios with little cross-sectional applicability.

An alternative method is Continuous Approximation (CA). CA approaches describe the travel demand and decision variables as scalar variables or continuous functions over a geographical space (Ibarra-Rojas et al. 2015)[18]. A pioneering work was introduced by Vuchic and Newell (1968)[19] followed by many following works (Byrne 1975[20]; Chen et al. 2015, 2018[21,22]; Fan et al. 2018[23]; Ouyang et al. 2014[24]; Saidi et al. 2016[25]; Vaughan 1986[26]). Particularly on feeder transit design, Hurdle (1973)[27] developed a CA model to solve parallel feeder lines planning problem. The methods of elementary calculus were used to solve the optimization problem. Wirasinghe (1980)[28] optimized the perpendicular feeder bus lines to a rail line in a rectangular area. Further, Kuah and Perl (1988)[29] simultaneously designed feeder-bus route spacing and stop spacing. Chien and Schonfeld (1998)[30] extended the solely feeder bus design problem to a joint design problem of both the rail transit line and its feeder bus routes. Such a trunk-feeder joint design problem was recently extended by Fan and Mei (2017)[31] with more sophisticated design to the feeder buses, of which the stop spacings were location dependent. Instead of only considering a single trunk line, Sivakumaran et al. (2014)[32] proposed an idealized network where trunk lines took the form of a rectangular grid and feeder-bus lines reside within the quadrant. However, the demand was assumed to be distributed uniformly over the city. The CA approach is also utilized by Sivakumaran et al. (2012)[33] for the schedule coordination of a trunk-feeder transit system. Using a continuous approximation model, Hugo Badia and Erik Jenelius (2021)[34] et al. investigated the effects of two feeder strategies for vehicle automation and electrification on the applicability of fixed routes and door-to-door services. Xin Li (2022)[35] based on an approximate grid-type road network and considering the various route choices of passengers, constructed a bi-level mixed-integer optimization model to optimize the urban rail transit and regular bus network. Jingyuan Qiao (2023)[36] used the continuous approximation method to optimize the strategy between conventional transit and demand in ring-radial cities.Table 1 provides comprehensive definitions of all variables and parameters used in the optimization model. It includes decision variables (*u*_*i*_(*x*) and *g*_*i*_(*x*)) and all input parameters such as station density, passenger demand, route length, walking speed, waiting cost, etc. Each parameter is accompanied by a detailed description, value/unit, and reference source, providing a comprehensive technical foundation for model construction.

Even though CA models can help find the optimal solution easily and efficiently, the design results obtained from CA models are not ready for implementation. Because CA models omit much of the real-world detail, such as street network, existing bus routes and land use patterns (Mekuria et al. 2012[37]; Sivakumaran et al. 2012[33]). CA models can play a critical role in putting forward cost-efficient and practical solutions to trunk-feeder design problem if they are complemented by implementation ready processes that can transition the theoretical design solutions to practice. Efforts had been devoted to enhancing the practicality of CA model-based designs, they have been limited to few studies. Wirasinghe and Ghoneim (1981)[38] proposed a method to locate optimal stops where the integral of stop density function yields an integer. Considering more realistic street layout, Chien et al. (2003)[39] transformed the irregular grid street network (including diagonal streets) to a pure grid transit network, in which feeder bus routing problem was formulated. To convert theoretical results to practice, another key factor is that the feeder bus routes must be aligned to operate in real streets. Given land use patterns and population distribution, Huang et al. (2010)[40], Simard et al. (2011)[41] solved the bus network optimization problem based on Geographic Information System (GIS).

**Fig 1:**
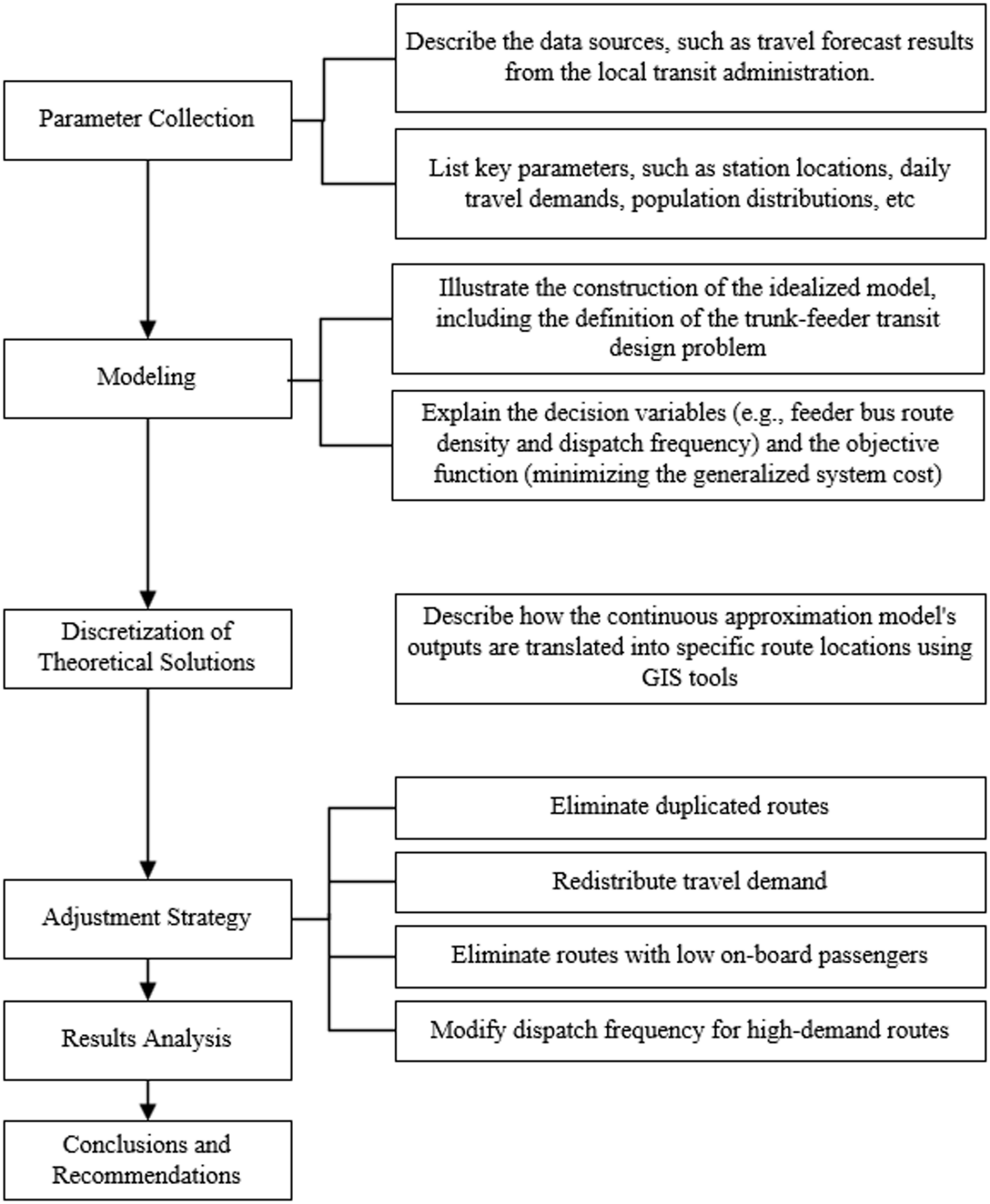
Key Steps in Feeder Bus Service Design Research.

To summarize, previous works have proposed models to design feeder network considering trunk line layout or jointly design trunk-feeder system. In this paper, the research area is an urbanized corridor where it is appropriate to formulate input parameters and decision variables as smooth, continuous functions. Thus, we could take advantage of the continuum approximation model to help obtain more general insights as is widely used in literature. To avoid the weak points of CA model, we furtherly combine the GIS tool with the theoretical results and offer implementation-ready designs. The rest of the paper is organized as follows. Section 2 describes the process of modeling in detail, and the method of integrating the density is applied to find the exact location of each feeder bus route. In Section 3, an adjustment process is proposed to fine-tune theoretical results based on realistic street map and other built environment characteristics with GIS. Section 4 analyzes the final results and demonstrates the effectiveness of the adjustment. In addition, other practical suggestions are proposed for the sake of all participants. The last section concludes the study. Fig 1 illustrates the key steps in this feeder bus service design research.This figure presents the complete methodology and workflow of the feeder bus service design research. It illustrates the key steps from theoretical modeling to GIS-assisted adjustments, including data collection, optimization model construction, initial solution generation, and the four-step adjustment strategy based on real-world conditions. This flowchart helps readers comprehensively understand the overall structure and methodological framework of this study.

## 2 Methodology

To design the feeder bus services for the pre-determined trunk line, The Line, the paper takes advantage of the existing CA model by Wirasinghe (1980)[28] and Fan and Mei (2017)[31] to prepare demand data and identify the optimal route densities and frequencies of feeder bus services. The objective is to minimize the generalized system cost, which is the sum of user cost and operator cost.

### 2.1 The idealized the line layout

The line is an east-west light rail line with 21 stations, connecting to Metrorail lines, Regional commuter rail lines, National Railway, and local bus routes. It also serves major economic centers and connects people to jobs. The line separates the corridor to north and south sides. In the following, we consider two sides separately.

**Fig 2:**
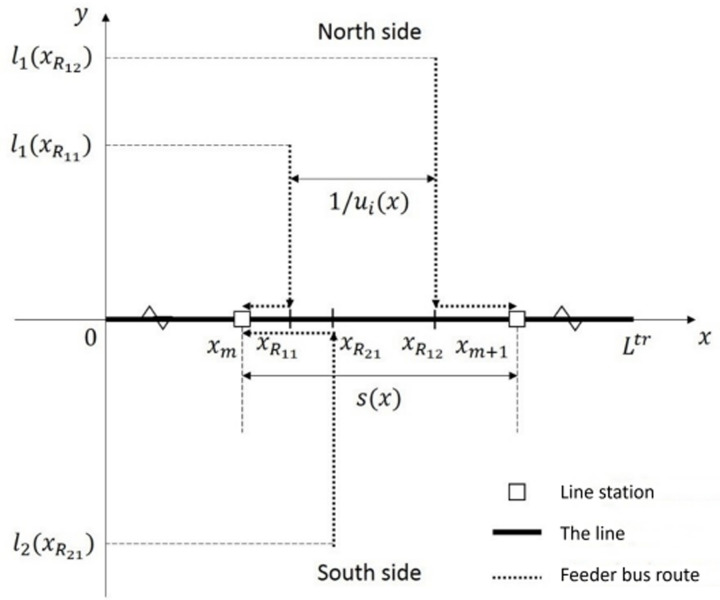
Trunk-feeder framework along idealized linear corridor (xm,xm+1 $x_{m},\;x_{m+1}x_{m},\;x_{m+1}$ **: the location of the line station; xR11,xR21,xR12** $x_{R_{11}},\;x_{R_{21}},\;x_{R_{12}}$ **: the location of feeder bus location).**

Without loss of generality, the line is idealized into a linear corridor with length *L*^*tr*^. By design, there are 21 planned stations with density *1/s*(*x*) as a function of location *x*. Inter-station feeder bus lines are aligned to be perpendicular to line at two sides, denoted by subscript *i* = 1,2. The feeder bus density (i.e., the inverse of route spacing) for side iis denoted by $u_{2}(x)$as a function of location *x*. Upon reaching line they run parallel to the closest station. By observing geographical conditions, we define the service boundaries of feeder bus service as$l_{i}(x)$ as a continuous function of location *x*. Fig 2 illustrates the trunk-feeder framework along idealized linear corridor.This figure illustrates the trunk-feeder framework model along an idealized linear corridor. It displays the spatial relationship between the rail transit line (trunk) and the perpendicular feeder bus routes connecting various stations. Key elements include the rail station locations (*x*_*m,*_*x*_*m*+1_) and the locations of various feeder bus routes (*x*_*R*11_, *x*_*R*21_, *x*_*R*12_, etc.). This figure is essential for understanding the geometric layout and spatial relationships in the model.

### 2.2 Assumptions

Like some existing literature, following assumptions are made to facilitate the modeling.

1. According to the research results from Fan and Mei (2017) [31], the feeder bus stop density varying along y-axis brings only less than 0.4% benefit as compared to the simplified uniform-stop strategy. Thus, allocation of feeder bus stops is neglected in this paper.
2. Each station of the line has at least one dedicated feeder bus route. Feeder bus routes are perpendicular to x-axis (The line) and turn to the closest rail station without stopping when reaching the line.
3. Passengers would choose the closest bus routes without considering waiting time and bus speed. And they need to transfer to the The line for the line-haul portion of the trip.
4. The capacity of the feeder bus is sufficient to take all the passengers waiting for the bus. Passengers do not have to wait for more than one bus.
5. High-frequency service of the Line (12 trains/hour during peak hours) renders passengers’ negligible transfer waiting time (according to the local transit administration’s 2022 data).

### 2.3 Optimization model

#### 2.3.1 Variables and parameters.

The density of feeder bus routes and dispatch frequency are the decision variables in the model. The input data of travel demand is obtained from Local Transit Administration (2022) [42]. The feeder bus service boundary is measured from OpenStreetMap according to National Center for Smart Growth Research & Education [43]. Other parameters are adopted from Chien et al. (2003) [39], Mekuria et al. (2012) [37], and Schmitt (2022) [1]. Table 2 defines all the variables and parameters involved in the model.

**Table 2:** Definitions of Variables and Parameters.

Definitions	Cite	Descriptions	Value/Units
Decision Variables			
$u_{i}(x)$	[2,3]	Density of feeder bus routes in the vicinity of x on side i	#/km
$g_{i}(x)$	[2,3]	Dispatch frequency of feeder bus routes in the vicinity of x on side i	#/hour
Parameters			
1/s(x)	[10,11]	Density of the line stations in the vicinity of x	–
$P_{i}(x)$	[33]	Approximate passenger demand per unit time taking a bus to the line stations in the vicinity of x on side i	2040 forecast data
$L^{tr}$	[32]	Length of the the line	26.3 km
$l_{i}(x)$	[32]	Length of the feeder bus routes in the y-direction in the vicinity of x on side i	–
v	[6]	Average speed of feeder bus	16 km/h
w	[6]	Average speed of walking	4.32 km/h
$\gamma_{A}$	[39,40]	Average cost of walking per unit time per passenger	$12/pass-h
$\gamma_{B}$	[39,40]	Average cost of operating a feeder bus per unit time per vehicle	$143/veh-h
$\gamma_{R}$	[39,40]	Average cost of riding on a bus per unit time per passenger	$6/pass-h
$\gamma_{W}$	[39,40]	Average cost of waiting for a bus per unit time per passenger	$10/pass-h

#### 2.3.2 Constructing the total cost.

The trunk-feeder system is optimized in the x-direction in terms of user cost and operator cost considering different demand on both sides of the line.

User cost includes: (i) the cost of walking to the bus route *C*_*A*_; (ii) the cost of waiting for a bus *C*_*W*_; (iii) the cost of travel-time when passengers are riding on a bus$C_{r}^{fd}$. Operator cost is the cost of operating the feeder bus vehicles from the perspective of the operator,$C_{o}^{fd}$. The total cost *C*C is constituted by the above four parts and expressed as follows:


$$C=C_{\left(A\right)}+C_{\left(W\right)}+C_{\left(r\right)}^{\left(fd\right)}+C_{\left(o\right)}^{\left(fd\right)}$$ (1)

On average, passengers’ walking distance is 1/4*u*_*i*_(*x*) in the x-direction on the side *i*i. Thus, the total cost of walking to the bus route per unit time is expressed as follows:


$$C_{A}=\sum_{i=1}^{i}\left[\gamma_{A}*P_{i}(x)/4wu_{\left(i\right)}(x)\right]$$ (2)

The average waiting time for a passenger is equal to one half the headway. Thus, the total cost of waiting for a bus per unit time is expressed as follows:


$$C_{W}=\sum_{i=1}^{2}\left[\gamma_{W}*P_{i}(x)/2g_{i}(x)\right]$$ (3)

The average riding distance in the x-direction is s(x)/4. Thus, the riding time for passengers on a bus in the x-direction is s(x)/4v. The total cost of riding for passengers per unit time is:


$$C_{r}^{fd}=\sum_{i=1}^{2}\left[\gamma_{R}*P_{i}(x)*s(x)/4v\right]$$ (4)

The average distance traveled by bus in the x-direction is equal to the average riding distance in the x-direction, and the distance traveled by bus in the y-direction is equal to the boundaries in the y-direction, which varies according to the terrain and street geometry. Therefore, the return trip length for the feeder bus route in the vicinity of *x* is $2[l_{i}(x)+s(x)/4]$. The number of buses dispatched per unit time in the vicinity of x on the side *i* is *u*_*i*_(*x*)*g*_*i*_(*x*). Consequently, the total cost of operating the feeder buses per unit time is given by:


$$C_{o}^{fd}=\sum_{i=1}^{2}2\gamma_{B}[l_{i}(x)+s(x)/4v]\;u_{i}(x)g_{i}(x)$$ (5)

#### 2.3.3 Objective function and constraints.

Based on the above constructed cost terms, the objective function (Equation 6a) is to minimize the total system cost with respect to the decision functions *u*_*i*_(*x*) and *g*_*i*_(*x*).


$$minC=\sum_{i=1}^{2}\{\gamma_{A}*\frac{P_{i}(x)}{4wu_{i}(x)}+\gamma_{W}*\frac{P_{i}(x)}{2g_{i}(x)}+\gamma_{R}*P_{i}(x)*\frac{s(x)}{4v}+2\gamma_{B}\left[l_{i}(x)+\frac{s(x)}{4v}\right]u_{i}(x)g_{i}(x)\}$$ (6a)

In the model, the number of passengers on each bus cannot exceed the bus capacity. According to Chien et al. (2003) [39], the bus capacity is 50 passengers per vehicle. Meanwhile, considering the utility and feasibility of feeder bus, each bus is assumed to take at least 5 passengers. According to Salek and Machemehl (1999) [44], the average waiting time is equal to half the headway when the headway is under 30 minutes, so the minimum dispatch frequency of feeder bus is set to 2 vehicles per hour. According to Walker (2011) [45], the maximum accepTable walking distance for the line feeder bus riders is around 600 meters. Combined with the current situation of bus operation, the density of feeder bus routes is set between 0.8 and 2 per kilometer. Thus, the objective function is subject to:


$$5\leq \frac{P_{i}(x)}{u_{i}(x)}*\frac{1}{g_{i}(x)}\leq 50$$ (6b)

$$g_{i}(xge2$$ (6c)

$$0.8\leq u_{i}(xle2$$ (6d)

In this case, we have a nonlinear constraint and bound constraints. The model is solved by using a nonlinear programming solver in MATLAB.

### 2.4 Input parameter descriptions

**Fig 3:**
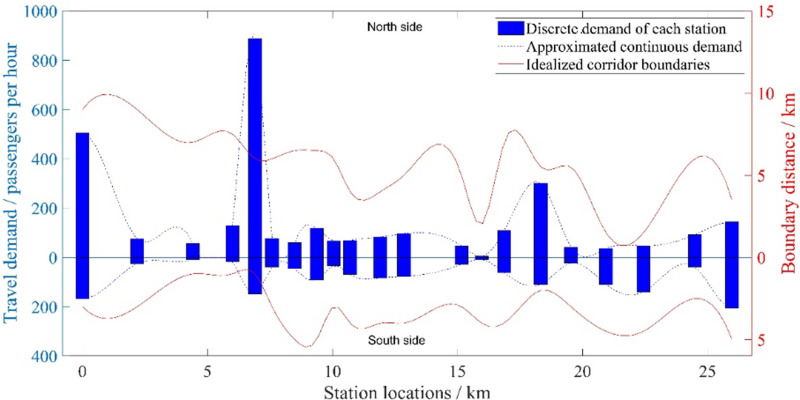
Travel demand along the corridor.

Travel Forecasts Results Technical Report (Local Transit Administration 2022) provides the line’s station locations and corresponding daily travel demand of each station. We convert the daily demand of each the line station to peak-hour demand *P*_*i*_(*x*) by multiplying the peak-hour parameter 20%. The complete raw dataset used for this analysis is provided in S5 Table, which contains all original station-level data and serves as the foundation for our model development. As shown in Fig 3, the (blue) bars denote the discrete hourly travel demand of each station and are fitted to (blue) dashed curves via Spline function in MATLAB, which indicate the continuum approximated demand along the corridor. The (red) curves show the coverage boundaries at two sides of the corridor.

### 2.5 Locations of feeder bus routes

In theory, the feeder bus routes may be located at the set of points$\left\{R_{j}\right\}$where the integral of density of feeder bus *u*_*i*_(*x*) is equal to an integer (Equation 7). However, the exact location in practice could differ a little in the vicinity of the theoretical location to match the realistic street map.


$$\int_{0}^{R_{j}}u_{i}(x)dx=m\;\;m=1,2,3,...$$ (7)

**Fig 4:**
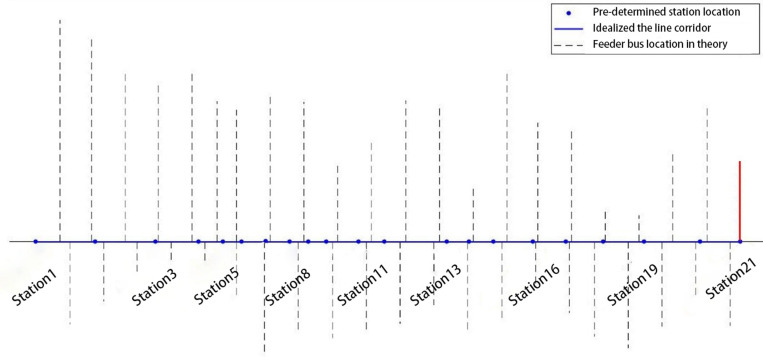
Optimal locations of feeder bus routes in theory.

It is worth noting that the integral over the entire line length may not happen to yield an integer. So, at the edge of the line (near the last station 21), we set a specified tolerance 0.05 to determine whether there is another feeder bus route for the last station. Specifically, if the difference between the integral and the nearest integer is less than 0.05, there should be a feeder bus route at the last station. In this case, the integral of density on the north side over the entire line length is 21.98, which means the 22^nd^ bus route should exist at the last station within the tolerance. Nevertheless, the integral of density does not meet the tolerance for the south side, whose value is 21.30. So based on the initial results, an additional feeder bus on the north side should be added manually to serve station 21(the red solid line shown in Fig 4), as it is needed to extend the service area at the end of the line. Totally there are 22 feeder bus routes on the north side and 21 routes on the south side. Fig 4 shows the relative locations of this trunk-feeder system provided by the model outputs. It presents the optimal distribution of feeder bus route locations calculated by the theoretical model, indicating the theoretical positions of 22 feeder routes on the north side and 21 routes on the south side. A red solid line specifically marks an additional feeder route on the north side that needs to be manually added to Station 21. This visual representation of the optimization model output shows the theoretically optimal feeder network layout before adjustments.

### 2.6 Route location adjustment

The initial solution from optimization satisfies the assumptions and constraints, which means the theoretical model is feasible. To apply it to reality, a planner would start with the initial results to modify the locations based on complex real-world conditions and their experience and knowledge of the system. GIS is utilized to modify the theoretical results in this study. The most suiTable location for feeder bus routes should be located near a populated community and avoid duplication with existing bus routes. We use the Spatial Analyst tool to create raster surfaces first. Euclidean distance is calculated for each existing bus stops, and the population of each census tract is visualized. Then the two raster surfaces are reclassified and combined to show the value of suitability of feeder bus location.

Step 1: Eliminate duplicated routes. Firstly, we focus on stations served by more than one feeder bus route. If the realistic population is low along the route or a bus route already exists, we delete a duplicated route according to the street map. For example, on the south side near Station 3, there are two feeder bus routes nearby in theory. One is on its left side, and the other is on the right. But according to the street map, the left one could match a road better. Thus, the right one is deleted. In another example, a redundant route between Station 19 and Station 13 is eliminated as Metro buses F4 and F6 could serve the community nearby. In this step, two routes on the north side and two on the south side are eliminated.

Step 2: Redistribute travel demand. According to the locations of the line stations and feeder bus routes, the station which a feeder bus route serves is determined. Then the travel demand for each bus route can be determined and redistributed rather than using the approximate continuum demand function in the model. For example, there is no feeder bus route for Station5 on the south side. Because the station’s location is very close to the boundary of D.C., it is too narrow to add a feeder bus route. The travel demand could be satisfied by existing bus routes J1, J2 and J4. In another example, the feeder bus route between Station8 and Station9 could be shared due to the small spacing (less than 700 meters). Passengers would still choose the nearest bus route.

Step 3: Eliminate the routes with low on-board passengers. According to the redistributed travel demand after Step 1–2, recalculate the average on-board passengers on each vehicle of the remained feeder bus routes. The average on-board passengers of some routes may become less than 5 persons per vehicle. Those routes are deleted to decrease the operator cost. In this step, one route on the north side and three on the south side are deleted. The travel demand along these routes could be satisfied by existing bus routes.

Step 4: Modify the dispatch frequency for some high-demand routes. After Step 3, the average on-board passengers on some feeder bus routes may exceeds the vehicle capacity, 50 persons. Accordingly, the dispatch frequency or vehicle type of these routes should be modified to increase the capacity during the time period.

Finally, the adjusted feeder design includes 19 routes on the north side and 16 routes on the south side.

## 3 Results analysis and discussions

Table 3 presents the final designs adjusted from the initial solutions of the theoretical model. Station5 is the station with the heaviest travel demand. According to the results, the optimal headway of the feeder bus for the station is three minutes to meet the travel demand, which means 20 buses in an hour with 49 passengers on each vehicle averagely.

**Table 3:** Final design after adjustment process.

The north side of the line			The south side of the line		
Feeder bus destination station	Peak-hour headway (min)	Average vehicle load (passengers)	Feeder bus destination station	Peak-hour headway (min)	Average vehicle load (passengers)
Station1	6	50	Station1	15	42
Station2	30	37	Station2	30	12
Station3	30	28	Station5	20	12
Station4	20	43	Station7	30	22
Station5	3	49	Station8	20	41
Station6	10	12	Station10	20	22
Station7	30	29	Station11	20	27
Station9	15	46	Station12	30	38
Station10	20	23	Station13	30	13
Station11	20	27	Station15	20	20
Station12	20	32	Station16	15	27
Station13	30	23	Station17	30	11
Station15	12	21	Station18	20	36
Station16	10	50	Station19	15	34
Station17	30	20	Station20	30	19
Station18	20	12	Station21	12	41
Station19	20	15			
Station20	20	30			
Station21	15	36			

Table 4 summarizes the before-and-after comparison results in terms of hourly cost (for user, operator and total). It is observed that the total cost saving of adjustment will be 3.5%. Although the user cost is increased by 5.2%, the benefit in the supply side outweighs the loss in the user cost. After the adjustment process, 95% of patrons walk less than 500 meters on average, and the rest walk less than 800 meters. Considering the existing bus services, the loss of the user cost should be mitigated than the conservation estimation in our model, where patrons’ walk distance was measured to new feeder bus routes only

**Table 4:** Results comparison.

	Initial results	Modified results	Percentage change
User Cost ($)	10700	11254	5.2%
Operator Cost ($)	12275	10913	-11.1%
Total Cost ($)	22975	22167	-3.5%

**Fig 5:**
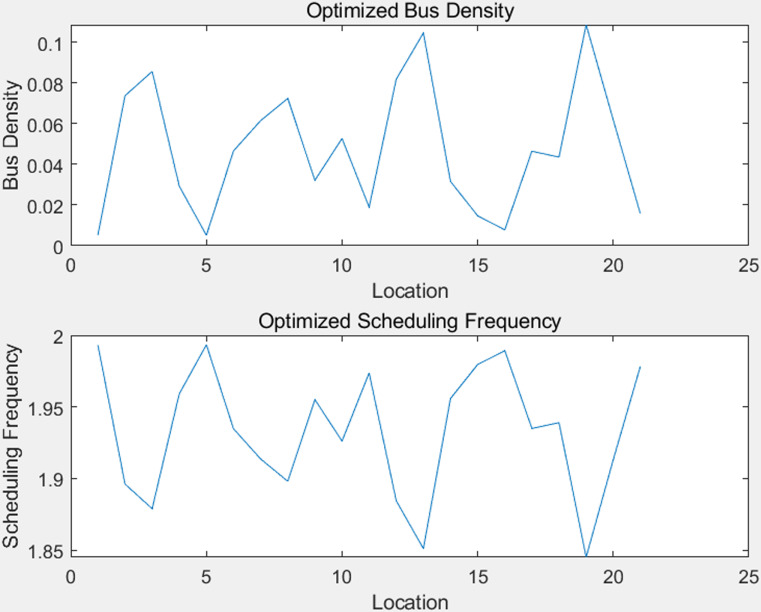
Optimized Bus Density and Scheduling Frequency.

**Fig 6:**
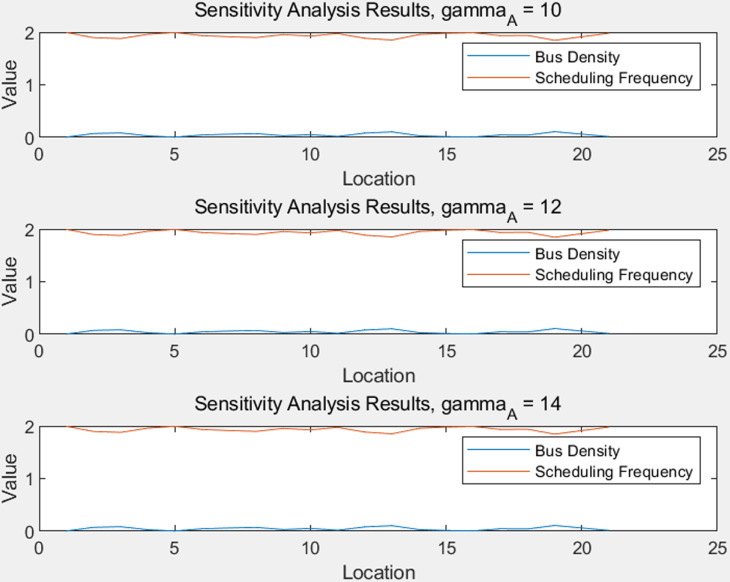
Sensitivity Analysis Results for Different Gamma Values.

Fig 5 and Fig 6 illustrate the variations in the optimized bus density and dispatch frequency, as well as the results of the sensitivity analysis.

In Fig 5, the top sub-figure shows the variation in bus density at different locations. The title is “Optimized Bus Density,” with the horizontal axis representing the location and the vertical axis representing the bus density. It can be observed from the figure that there are significant fluctuations in bus density across different locations, indicating that the distribution of buses is not uniform. These fluctuations may be due to the varying demand for buses in different areas. Through optimization, these densities have been adjusted to improve the overall efficiency of the system.

The bottom sub-figure shows the changes in dispatch frequency. The title is “Optimized Dispatch Frequency,” with the horizontal axis representing the location and the vertical axis representing the dispatch frequency. It can be seen from the figure that there are also noticeable changes in dispatch frequency across different locations. This indicates that dynamic adjustments to bus dispatching were made during the optimization process to better meet the demand at various locations. Such optimization helps to improve the utilization efficiency of buses, reduce waiting times, and lower operating costs.

In Fig 6, the sensitivity analysis results under different gamma_A values (10, 12, 14) are presented. Each sub-figure is titled “Sensitivity Analysis Results, gamma_A = 10,” “Sensitivity Analysis Results, gamma_A = 12,” and “Sensitivity Analysis Results, gamma_A = 14,” respectively. The horizontal axis represents the location, and the vertical axis represents the value, with the legend including two curves for “Bus Density” and “Dispatch Frequency.”

From these sub-figures, it can be seen that regardless of the value of gamma_A, the curves for bus density and dispatch frequency are relatively stable. This indicates that the optimization results maintain a high level of stability under different sensitivity parameters, and the optimization method has good robustness. Through sensitivity analysis, it can be seen that the optimization scheme is not sensitive to parameter changes, indicating that the optimization model has strong adaptability and stability.

In summary, the optimized results show the dynamic adjustment of bus density and dispatch frequency, which helps to improve the overall performance of the system. At the same time, the sensitivity analysis has verified the robustness and stability of the optimization method, indicating that the method has good reliability in practical applications. Such optimization analysis is of great significance for improving the efficiency and service quality of public transportation systems.

The feeder bus service design approach proposed in this study demonstrates several advantages over the existing system, particularly in terms of implementation and operational costs, as well as time efficiency. Here is a detailed comparison between the proposed design and the current system:

### 4.1 Implementation costs

The existing feeder bus system may have established operational patterns and infrastructure investments. In contrast, the proposed design allows for more flexible adjustments to routes and service frequencies to adapt to changing passenger demands. Although initial adjustments may incur some costs, the long-term flexibility can reduce waste due to mismatched supply and demand.

### 4.2 Operational costs

By optimizing route density and service frequency, the proposed design reduces inefficiencies in operation, leading to lower fuel consumption and maintenance costs. Additionally, better route planning minimizes driver working hours, thereby reducing labor costs.

### 4.3 Travel time for passengers

The optimized feeder bus service design takes into account passenger convenience, significantly reducing total travel time by minimizing transfer frequency and walking distance to stations. According to the adjustment strategy in this study, 95% of passengers walk less than 500 meters on average, with the remainder walking less than 800 meters, greatly enhancing the passenger experience.

### 4.4 Service coverage and accessibility

The new design improves service coverage and accessibility through more rational route layouts and service frequencies, attracting new passenger groups and increasing the attractiveness of the public transit system.

### 4.5 Time efficiency

By reducing passenger waiting times and vehicle operation times, the new design enhances the time efficiency of the entire transit system. For example, for the station with the highest demand, the feeder bus service interval is reduced to three minutes, significantly cutting down waiting times.

### 4.6 Environmental impact

More efficient operation strategies reduce idling and congestion, thereby lowering greenhouse gas emissions and air pollution, positively affecting the environment.

### 4.7 Social benefits

The optimized feeder bus service design enhances community connectivity and promotes social inclusiveness by providing faster and more convenient transportation services, especially for areas with poor transit access.

### 4.8 Economic benefits

Although some initial investment may be required to adjust existing infrastructure and operational models, the reduced operational costs and increased passenger volume will bring economic benefits to transit operators in the long run.

Through the above comparison, it is evident that the feeder bus service design approach proposed in this study outperforms the existing system in terms of cost-effectiveness, time efficiency, and service quality. Future research can further explore the specific manifestations of these advantages in different urban and transit environments.

In real practice, many other measures may help further improve the designs. For instance, mix-sized vehicles can be operated rather than a single vehicle mode. In the feeder bus route with low load, micro-transit vehicles could be introduced, while routes with heavy load would favor larger-sized vehicles. Schedule coordination among new and existing bus lines is another promising measure to improve the system efficiency. Last but not least, bus route integration may also be possible for those closely located at both sides of the line to attract more potential patrons.

## 5 Conclusions

This paper combines a theoretical mathematical model with GIS tool to furnish practical designs of feeder bus services using a certain foreign light rail line as a case study. The theoretical model develops an initial solution first. To make the idealized solution adapt to the reality, an adjustment strategy is proposed with the application of GIS. By carefully observing local conditions, some feeder bus routes that overlap with existing bus routes are eliminated. Though route elimination will slightly increase the access cost of some passengers, it will bring considerable cost saving for the operators. Numerical examination indicates that the route and frequency adjustment brings over 3.5% total cost saving, and the saving of operation cost is even higher than 10%. The results also demonstrate the effectiveness and necessity of combining the theoretical model with a practical tool in transit design. The study of trunk-feeder system design along the line corridor highlights how decision makers can be informed by applying the state-of-the-art transit design models in practice.

This study combines theoretical mathematical models with geographic information system (GIS) tools to provide practical design for the feeder bus service design of specific light rail lines abroad. Although this study focuses on specific case studies, its methods and findings can provide valuable insights for other research directions in transportation planning and management, such as similar bus system design and intercity rail bus connections.Future extension may be directed to develop a decision-support tool that embeds together the CA design model and GIS tool to furnish an integrated platform for practitioners of any levels.

## Supporting information

S1 Table Definitions of Variables and Parameters. Comprehensive list of all variables and parameters used in the optimization model.
(TIF)

S2 Table Representative studies on feeder bus design problem. Summary of literature review comparing discrete models and continuum approximation models.
(TIF)

S3 Table Final design after adjustment process. Detailed information about the finalized feeder bus routes including headways and vehicle loads.
(TIF)

S4 Table Results comparison. Comparative analysis of system costs before and after the adjustment process.
(TIF)

S5 Table Original data. Complete raw dataset containing station locations, daily passenger volumes, and service area boundaries that served as the primary input for model development and analysis.
(TIF)
