# Supplementary figures and images for: Continuous approximation and GIS-enhanced design optimization of feeder bus networks along rail corridors
Source: PLoS One. 2025 May 23;20(5):e0318616. doi: 10.1371/journal.pone.0318616 (PMC12101712; doi:10.1371/journal.pone.0318616)

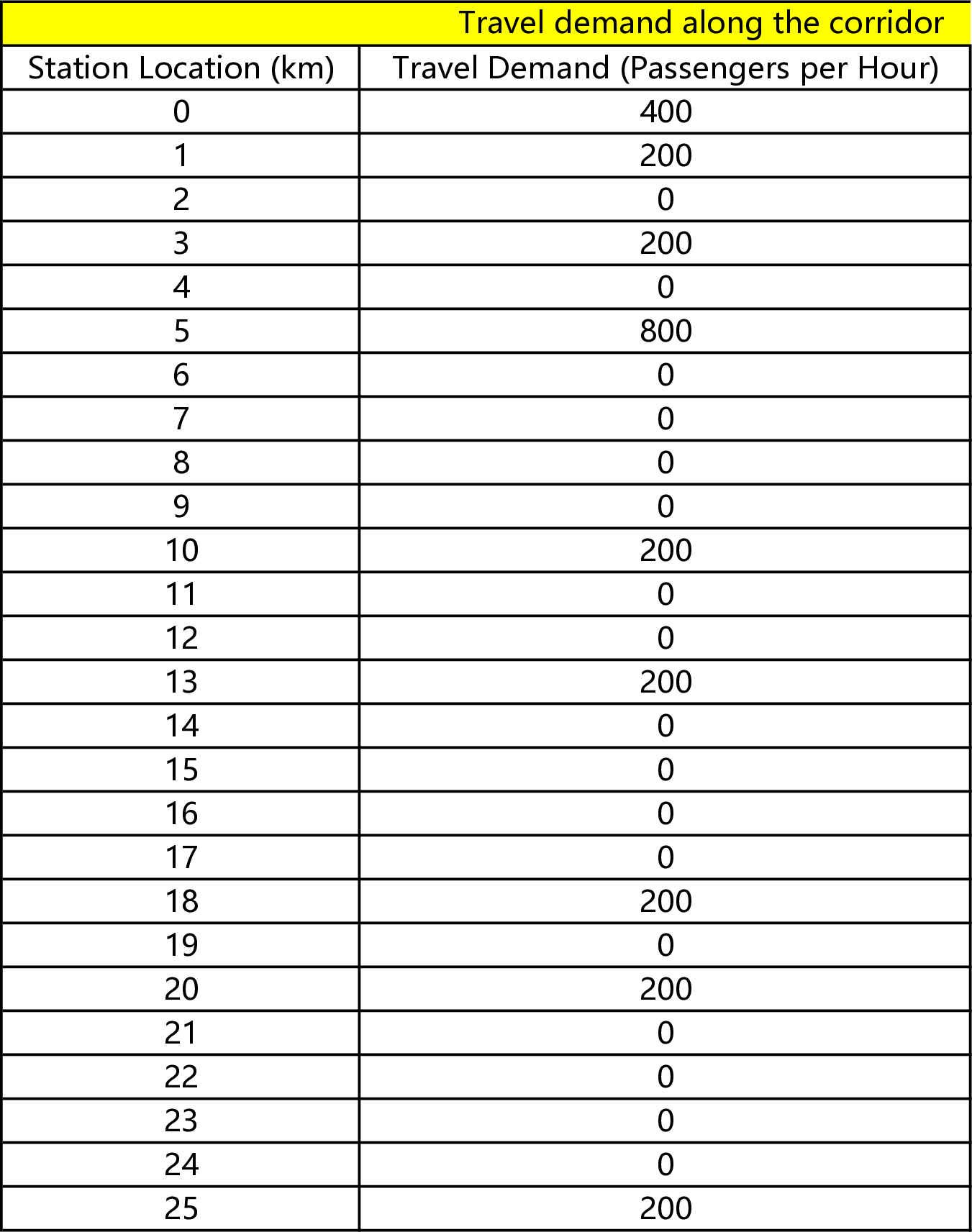

Supplement: S1 Table — Comprehensive list of all variables and parameters used in the optimization model. (TIF) [file pone.0318616.s001.tif]

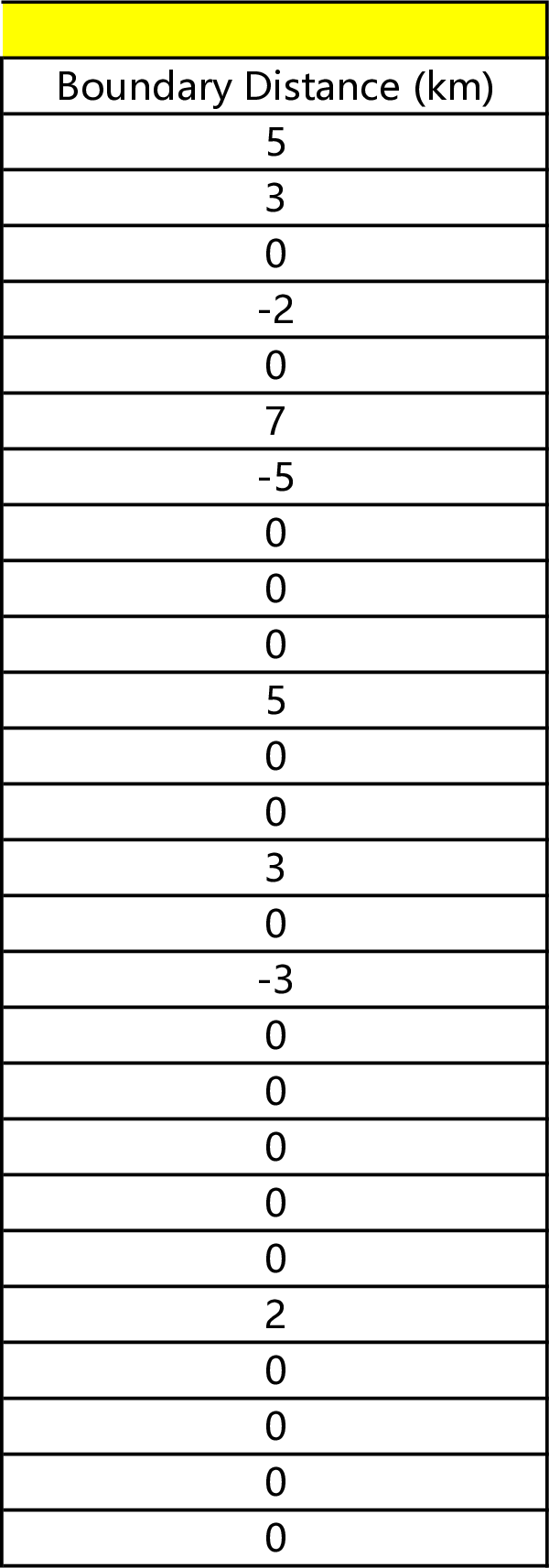

Supplement: S2 Table — Summary of literature review comparing discrete models and continuum approximation models. (TIF) [file pone.0318616.s002.tif]

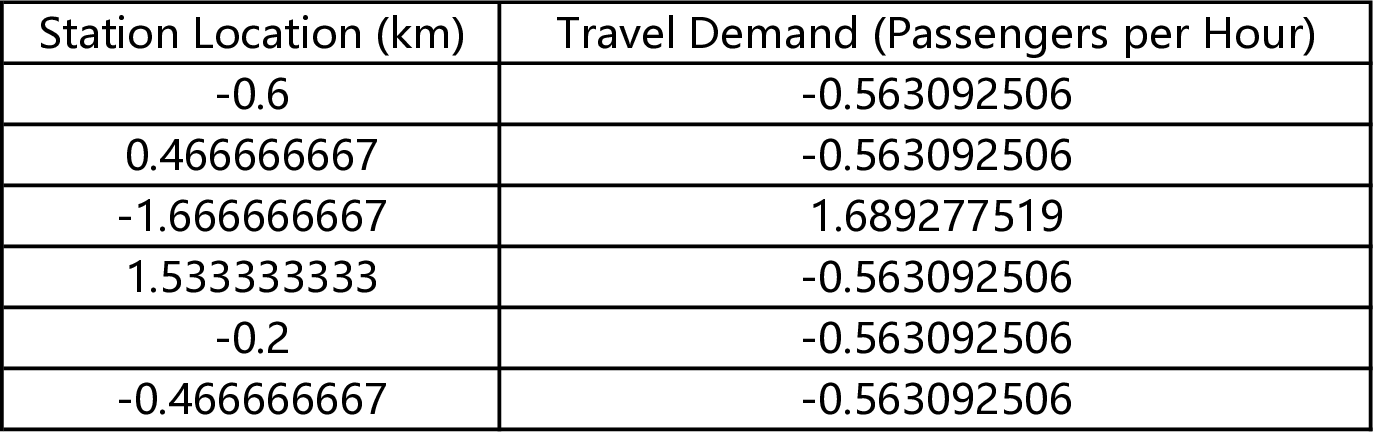

Supplement: S3 Table — Detailed information about the finalized feeder bus routes including headways and vehicle loads. (TIF) [file pone.0318616.s003.tif]

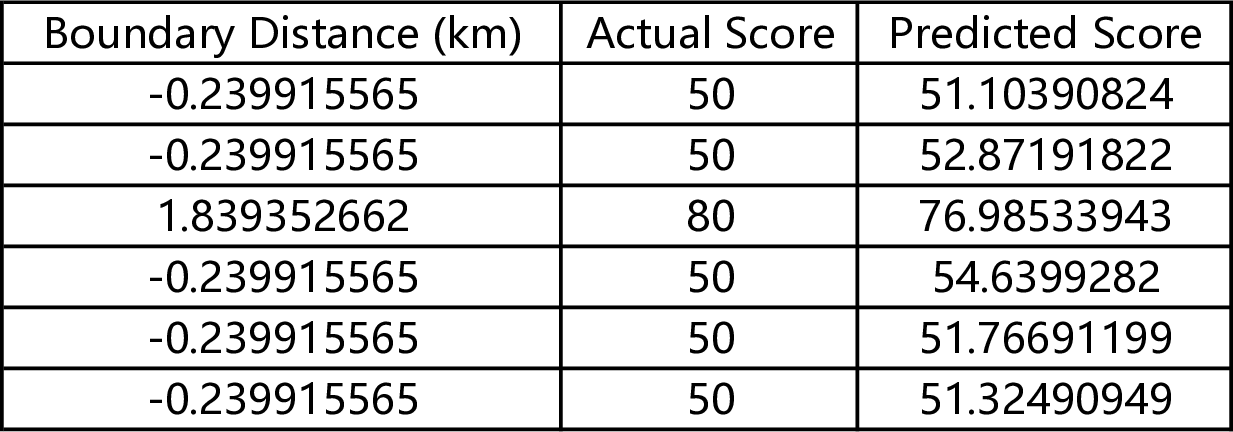

Supplement: S4 Table — Comparative analysis of system costs before and after the adjustment process. (TIF) [file pone.0318616.s004.tif]

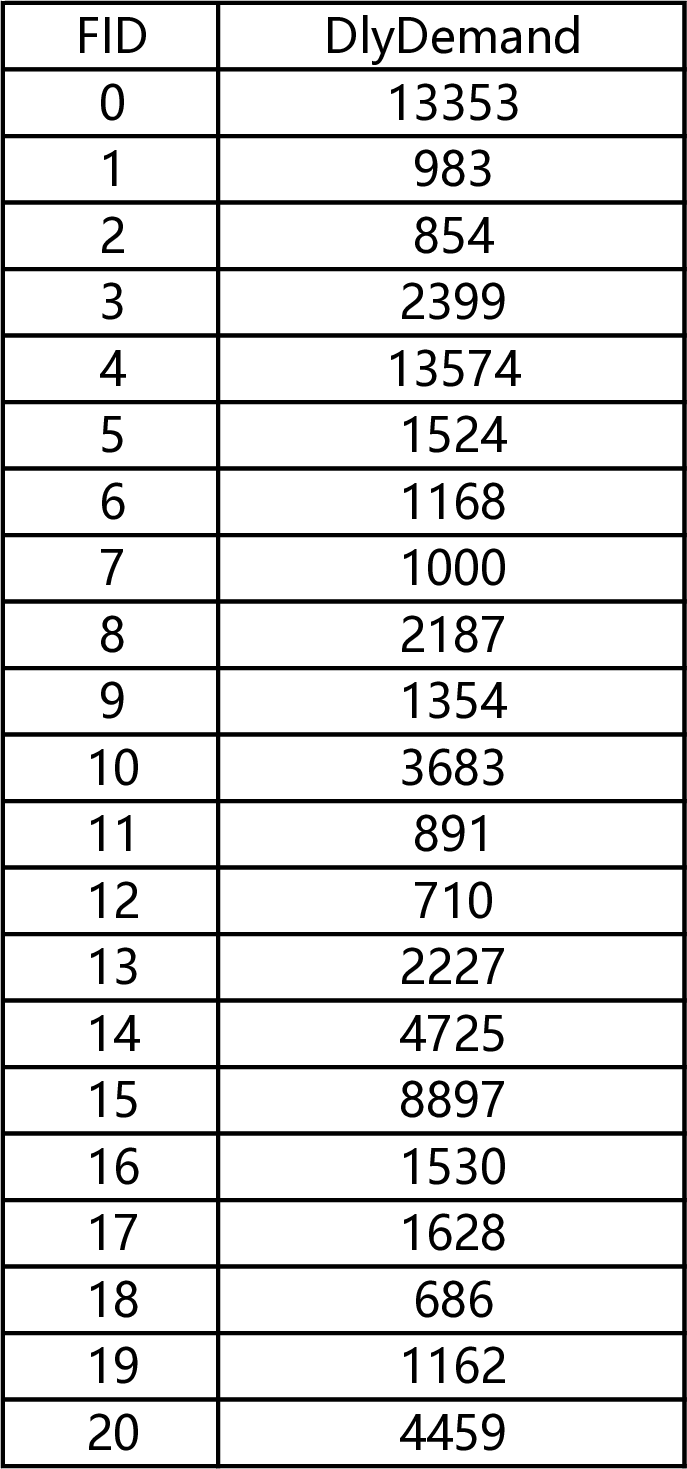

Supplement: S5 Table — Complete raw dataset containing station locations, daily passenger volumes, and service area boundaries that served as the primary input for model development and analysis. (TIF) [file pone.0318616.s005.tif]
